# Characterisation of the immune microenvironment of primary breast cancer and brain metastasis reveals depleted T-cell response associated to ARG2 expression

**DOI:** 10.1016/j.esmoop.2022.100636

**Published:** 2022-11-21

**Authors:** A. Giannoudis, D. Varešlija, V. Sharma, R. Zakaria, A. Platt-Higgins, P.S. Rudland, M.D. Jenkinson, L.S. Young, C. Palmieri

**Affiliations:** 1 Institute of Systems, Molecular and Integrative Biology, Molecular and Clinical Cancer Medicine, University of Liverpool, Liverpool, UK; 2 The School of Pharmacy and Biomolecular Sciences, RCSI University of Medicine and Health Sciences, Dublin, Ireland; 3 Department of Pathology, Royal Liverpool University Hospital NHS Trust, Liverpool, UK; 4 Department of Neurosurgery, The Walton Centre NHS Foundation Trust, Liverpool, UK; 5 Endocrine Oncology Research Group, Department of Surgery, RCSI University of Medicine and Health Sciences, Dublin, Ireland; 6 The Clatterbridge Cancer Centre NHS Foundation Trust, Liverpool, UK

**Keywords:** breast cancer, brain metastasis, TIL, immuno-oncology, ARG2

## Abstract

**Background:**

Immune checkpoint inhibition is an established treatment in programmed death-ligand 1 (PD-L1)-positive metastatic triple-negative (TN) breast cancer (BC). However, the immune landscape of breast cancer brain metastasis (BCBM) remains poorly defined.

**Materials and methods:**

The tumour-infiltrating lymphocytes (TILs) and the messenger RNA (mRNA) levels of 770 immune-related genes (NanoString™, nCounter™ Immuno-oncology IO360) were assessed in primary BCs and BCBMs. The prognostic role of ARG2 transcripts and protein expression in primary BCs and its association with outcome was determined.

**Results:**

There was a significant reduction of TILs in the BCBMs in comparison to primary BCs. 11.5% of BCs presented a high immune infiltrate (hot), 46.2% were altered (immunosuppressed/excluded) and 34.6% were cold (no/low immune infiltrate). 3.8% of BCBMs were hot, 23.1% altered and 73.1% cold. One hundred and twelve immune-related genes including PD-L1 and CTLA4 were decreased in BCBM compared to the primary BCs (false discovery rate <0.01, log2 fold-change >1.5). These genes are involved in matrix remodelling and metastasis, cytokine–chemokine signalling, lymphoid compartment, antigen presentation and immune cell adhesion and migration. Immuno-modulators such as PD-L1 (CD274), CTLA4, TIGIT and CD276 (B7H3) were decreased in BCBMs. However, PD-L1 and CTLA4 expression was significantly higher in TN BCBMs (*P* = 0.01), with CTLA4 expression also high in human epidermal growth factor receptor 2-positive (*P* < 0.01) compared to estrogen receptor-positive BCBMs. ARG2 was one of four genes up-regulated in BCBMs. High ARG2 mRNA expression in primary BCs was associated with worse distant metastasis-free survival (*P* = 0.038), while ARG2 protein expression was associated with worse breast–brain metastasis-free (*P* = 0.027) and overall survival (*P* = 0.019). High transcript levels of ARG2 correlated to low levels of cytotoxic and T cells in both BC and BCBM (*P* < 0.01).

**Conclusion:**

This study highlights the immunological differences between primary BCs and BCBMs and the potential importance of ARG2 expression in T-cell depletion and clinical outcome.

## Highlights

• BCBMs are immunologically more inactive than primary BCs.
• BCBMs have less TILs and reduced PD-L1 and CTLA4 transcript levels than primary BCs.
• Transcripts of PD-L1 and CTLA4 are higher in TN BCBMs as compared to other BCBM subtypes.
• ARG2 expression could be indicative of a depleted T-cell response in BCBM.
• ARG2 could be a potential marker of BC distant metastasis and a therapeutic target in BCBM.

## Introduction

Breast cancer brain metastases (BCBMs) are an increasing clinical problem in patients living with metastatic breast cancer (MBC).^1^ Therapeutic options for central nervous system (CNS) disease that progressed after local treatment are limited and remain an unmet clinical need.^1^

BC is a heterogeneous disease comprising several histological and molecular subtypes.^2^ These subtypes differ with respect to the tumour-infiltrating lymphocyte (TIL) component, the occurrence of immune evasive mechanisms and antigenicity.^3^^,^^4^ Triple-negative (TN) and human epidermal growth factor receptor 2 (HER2)-positive tumours contain higher TILs than estrogen (ER) and/or progesterone (PgR) receptor-positive BCs.^3^^,^^4^ ER-positive BC, in particular luminal A, is considered the least immunogenic since they have the lowest number of TILs and the lowest expression levels of tumour-associated antigens and neo-antigens.^3^^,^^4^

The predilection to metastasise to the CNS differs by BC subtype, with the incidence of BCBM ranging between 8% and 50%, with patients with HER2-positive and TN BC having the highest incidence.^5^, ^6^, ^7^, ^8^ BCBMs are known to differ at the histological and molecular level from their primary tumour.^8^^,^^9^ Data from Epidemio-Strategy-Medical-Economical (ESME)-MBC database demonstrated a discordance rate of 11.1% and 12.5% for ER and HER2, respectively, in BCBMs as compared to the primary BC.^9^ The presence of mutations and/or copy number alterations within BCBM that are absent in the primary breast tumour provides evidence of the distinct genomic landscape that exists within CNS disease.^10^^,^^11^ Differences in the immune tumour microenvironment have also been documented between primary and metastatic BCs, with MBC shown to have a lower TIL content and programmed death-ligand 1 (PD-L1) positivity compared to primary BCs as well as being more immunologically inert.^12^, ^13^, ^14^, ^15^, ^16^ Within these studies, BCBMs have been under-represented with only 3-21 cases included.^12^, ^13^, ^14^, ^15^, ^16^ A recent study of 93 paired primary BC and BCBM found fewer TILs in BCBMs with no differences observed with regard to PD-L1 expression by immunohistochemistry (IHC).^17^ Moreover, a gene expression network analysis of 58 BCBMs identified an immunosuppressed immune microenvironment and suggested several genes that could potentially serve as prognostic or therapeutic targets.^18^ An improvement in the understanding of the immune landscape in BCBM will help identify possible novel therapeutic targets and strategies as well as to enable stratification of BCBMs where an immune-oncology approach may be appropriate.

The aim of this study was to investigate how the immune landscape changes between the primary BC and their paired BCBMs and to identify BCBMs that will be potentially amenable to treatment with immune checkpoint inhibitors.

## Materials and methods

### Patients and samples

Fifty-five formalin-fixed paraffin-embedded (FFPE) samples consisting of 26 paired primary BCs and their BCBMs as well as three unpaired samples (one BC, two BCBM) were collected from the Liverpool Tissue Bank, Walton Research Tissue Bank (WRTB), Liverpool, UK and the Royal College of Surgeons Ireland (RCSI) National Breast Cancer Bio-resource, Ireland. The ER, PgR and HER2 status of the specimens was determined as previously described.^19^ The study was carried out in accordance with the Declaration of Helsinki and approved by the WRTB Ethics committee (WRTB15_06), the National Research Ethics Committee (NRES 11/WN003/2), the UK Health Research Authority (NRES 12/NW/0778) and the RCSI Institutional Review Board (#13/09; ICORG09/07). Appropriate approvals and written consent were in place before anonymised tissue and data were released.

### Tumour-infiltrating lymphocyte assessment

TILs were assessed by an experienced breast pathologist (VS) on haematoxylin–eosin (H&E) sections of the primary and metastatic tumours in accordance with the guidelines of the International TILs Working Group^20^ and defined as the percentage of infiltrating lymphocytes in tumour stroma within the boundary of the invasive tumour. Following TIL assessment, the immune response was further classified using the immunoscore described by Galon and Bruni^21^ as cold (absent), altered (excluded; TILs confined to peritumoural stroma), altered (immunosuppressed; TILs in intratumoural stroma) or hot presenting with a high TIL infiltrate. Based on the proportion of TILs and immunoscore, tumours were classified as follows: (i) cold/absent (low immunoscore or tumour with no or minimal immune cells) defined as 0%-10% of TILs present; (ii) altered defined when 10%-40% stromal TILs were present and (iii) hot (high immunoscore or tumour with high immune infiltrate) defined as >40% stromal TILs.^20^^,^^21^

### mRNA expression analysis

For the immune gene expression analysis, RNA was extracted using the miRNeasy FFPE Kit (Qiagen, Manchester, UK) and quantified on the ND-Nanodrop1000 spectrometer (ThermoFisher Scientific, Wilmington, MA). RNA integrity number was determined using the 2100 Agilent Bioanalyzer (Agilent Technologies, Palo Alto, CA). Profiling was carried out using the NanoString™ (Seattle, WA) nCounter™ IO360 Expression Assay [Human_v4 messenger RNA (mRNA)] according to the manufacturer’s instructions. The raw data were quality control assessed and normalised by the NanoString™ nSolver™ analysis software following the manufacturer’s recommendations. Three cases (BC712, BM912, BM1148) failed the NanoString™ normalisation and were excluded from downstream analysis. Therefore, analysis was carried out in 23 paired BC and brain metastasis (BM) cases and 6 unpaired samples and the normalised gene expression and differential expression (DE) analysis (nSolver™ 4.0 advanced analysis software) are presented in Supplementary Table S1, available at https://doi.org/10.1016/j.esmoop.2022.100636.

### ARG2 immunohistochemistry

The ARG2 protein expression was assessed by IHC in primary BCs and BCBM tissues using the rabbit monoclonal (ab264066) primary antibody (Abcam, Cambridge, UK). Nine BC cases that did not develop BCBM were also stained for ARG2. The ARG2 antibody was diluted 1/400 in phosphate-buffered saline with 1% bovine serum albumin (PBS/1% BSA) and incubated for 3 h after antigen retrieval in citrate buffer pH 6.0 (15 min microwave at full power followed by 15 min standing). The slides were washed with PBS and incubated for 30 min with the Envision+ System horseradish peroxidase (HRP)-labelled polymer anti-rabbit, followed by DAB+ Substrate Chromogen System for 10 min (DAKO/Agilent, Palo Alto, CA). The HRP-labelled polymer does not contain avidin or biotin and therefore, nonspecific staining resulting from endogenous avidin–biotin activity is eliminated. Prostate tissue was used as a positive control and the recombinant rabbit immunoglobulin G, monoclonal (SP137)-isotype (Ab1259830) antibody was used as a negative control (Abcam). We assessed the percentage of tumour cells and defined staining as negative when <1% of tumour cells expressed ARG2.^22^ The % staining was used for correlation with clinical outcome. Intensity was scored according to a four-tier system: 0, no staining; 1+, weak; 2+, moderate; and 3+, strong in order to calculate the H-score, a semi-quantitative measure of the staining intensity (0-3) multiplied by the percentage of positive cells (0%-100%). H-score was used to correlate protein to mRNA expression in our cohort as previously.^19^

### Statistical analysis

nSolver™ 4.0 advanced analysis software utilising the R3.3.2 plugins (cran.r-project.org) was used to normalise the data and carry out the principal component analysis (PCA), DE analysis, pathway scoring and gene-set enrichment analysis (GSA) following manufacturer’s recommendations. RAWGraphs (https://rawgraphs.io/) was used to generate the alluvial diagram illustrating the TIL change between primary BCs and BCBMs. The effect of ARG2 mRNA expression was assessed using the Kaplan–Meier (KM) plotter tool (www.kmplot.com),^23^ where distant metastasis-free survival (DMFS) is defined as the time between diagnosis of the breast cancer (BC) and the first metastatic site (lung, liver, brain). KM (log-rank) survival analysis was carried out for ARG2 protein expression (H-score), with breast–brain metastasis-free survival (BMFS) defined as the time between the initial breast surgery and the resection of the BM and overall survival (OS) defined as the time between breast diagnosis/surgery and death from any cause on GraphPad Prism v5.0 (GraphPad Inc, San Diego, CA). Wilcoxon signed rank *t*-test (Gaussian approximation) was used to compare the transcript levels of ARG2 with cytotoxic T cells, T cells and CD8 T cells.

## Results

### Tumour-infiltrating lymphocyte assessment

**Figure 1:**
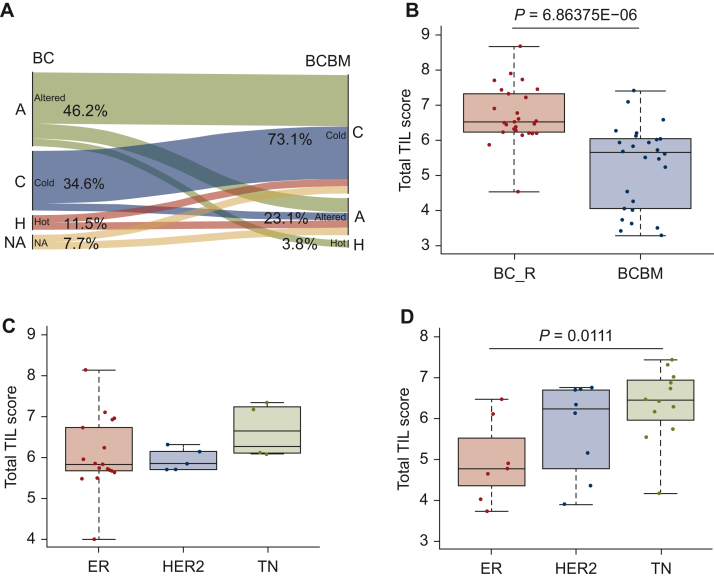
**Immunogenicity and tumour-infiltrating lymphocyte (TIL) score in BCBM.** (A) Immunological conversion of TILs between primary BCs and paired BCBMs. In primary BCs, 11.5% had a high immune infiltrate (hot; H), 46.2% were altered (immunosuppressed or excluded; A) and 34.6% were cold (C) tumours with low immune infiltrate. NA, not available tissue for scoring. In BMs, only 3.8% had a high immune infiltrate (H), 23.1% showed an altered-immunosuppressive profile (A) and 73.1% were cold (C) showing no or minimal immune infiltrate. (B) TIL immunoscoring showed a reduction of TILs in BCBMs in comparison to the primary BCs that relapsed to the brain (BC_R). **(**C) In the primary BCs, TIL immunoscore was higher in the TN subtype, with no difference between the ER-positive and HER2-positive subtypes. (D) In BCBMs, lower TIL immunoscore was observed in the ER-positive subtype and higher in the TN. BC, breast cancer; BCBM, breast cancer brain metastasis; BM, brain metastasis; ER, estrogen receptor; HER2, human epidermal growth factor receptor 2; TN, triple negative.

Assessment of TILs was carried out on H&E sections from 27 primary BCs and 28 BCBMs, 26 of which were paired samples. The clinical characteristics of the samples and pathological TIL assessment are presented in Supplementary Table S1, available at https://doi.org/10.1016/j.esmoop.2022.100636. Of these samples, 2 of 27 primary BCs and 1 of 28 BCBMs were not assessable due to insufficient material. A significant reduction of TILs in BMs was observed in comparison to the 26 paired primary BCs with a median value of 5 versus 13.5, respectively (*P* = 0.021). Of the primary BCs, 3 of 26 (11.5%) demonstrated a high immune infiltrate, 9 of 26 (34.6%) were altered-immunosuppressed, 3 of 26 (11.5%) were altered-excluded [total number of altered tumours 12/26 (46.2%)] and 9 of 26 (34.6%) were cold tumours. Two samples of the 26 (7.7%) were not assessable. This profile changed in the BCBMs with only 1 of 26 (3.8%) having a high immune infiltrate, 6 of 26 (23.1%) showing an altered-immunosuppressive profile and 19 of 26 (73.1%) were cold tumours showing no or minimal immune infiltrate (Figure 1A). Similarly, TIL immunoscore from the nCounter™ advanced analysis showed a reduction of TILs in BCBMs in comparison to the 26 primary BCs that metastasise to the brain (BC_R) (*P* = 6.86E−06, Figure 1B). Differences in the TIL counts were identified based on receptor subtypes both in the primary BCs and in BCBMs. In primary BCs, TILs were higher in the TN group, with no significant difference between the ER-positive and the HER2-positive BCs (*P* > 0.05, median values 6.64, 5.83, 5.84, respectively, Figure 1C). A significantly higher TIL immunoscore was observed in the TN BCBMs in comparison to the ER-positive BCBMs (*P* = 0.0111, median values 6.43, 4.77, respectively, and 6.11 for HER2, Figure 1D).

### Principal component analysis and differential gene expression

**Figure 2:**
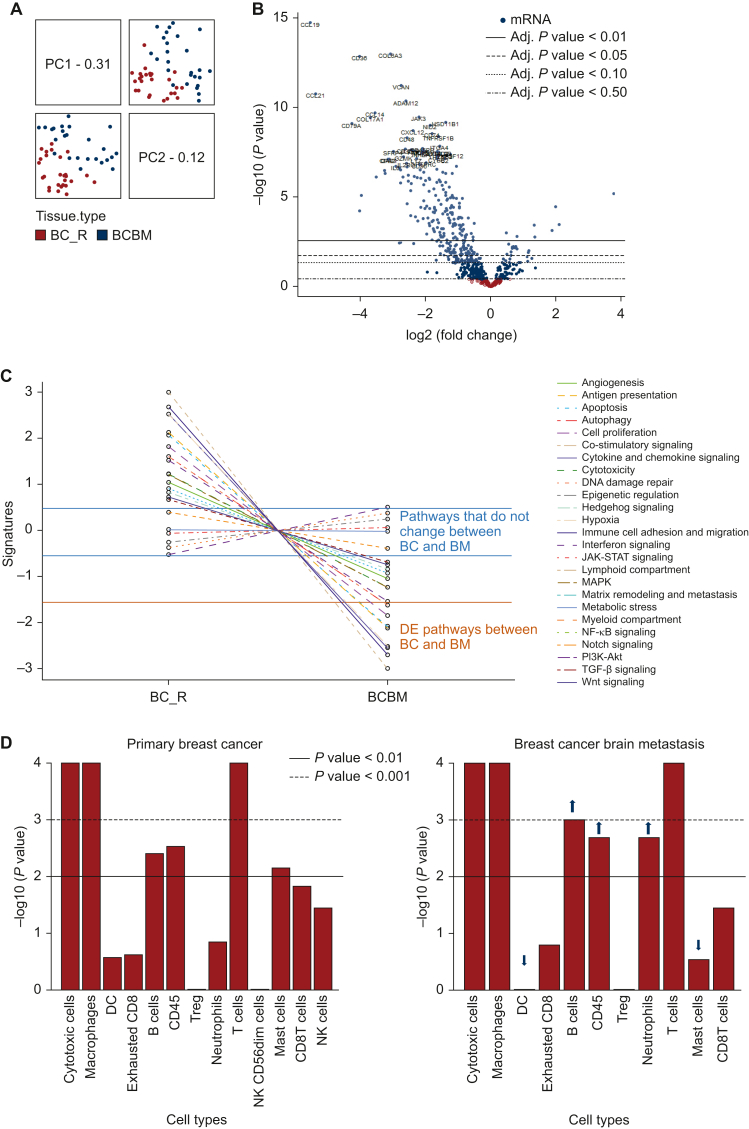
**Differential expression analysis of BCBM using the nCounter**™ **Advance Analysis software and immune cell type prevalence.** (A) Assessment of the global variation using principal component analysis (PCA) indicated a molecular distinction between the primary BCs that progress to brain metastasis (BC_R: red dots) and the BCBM (grey dots) cases, highlighting that differential expression is associated with cancer tissue type. (B) Two hundred and seventy-two DE genes were identified in BCBM versus primary BC (BC_R). Of these, 112 genes were down-regulated and 4 genes were up-regulated in BCBM with FDR <0.05 and log2 FC >1.5. (C) Dysregulated pathways in BCBM. (D) Prevalence of immune cell types in primary BCs that relapsed to the brain and in BCBMs showed lack of natural killer cells (NK and NK56dim:effector) and a reduction in dendritic cells (DCs) and mast cells in BCBMs, whereas there was an increase in neutrophils, B cells and CD45 cells highlighting the differences in the immune profile of BCBMs. The direction of immune cell type change is indicated by the black (lower prevalence) and grey (higher prevalence) arrows. BC, breast cancer; BCBM, breast cancer brain metastasis; DE, differentially expressed; FC, fold-change; FDR, false discovery rate; JAK-STAT, Janus kinases (JAKs), signal transducer and activator of transcription proteins (STATs); MAPK, mitogen-activated protein kinases; mRNA, messenger RNA; NF-κB, nuclear factor κB; PI3K-AKT, phosphoinositide 3-kinases and protein kinase AKT; TGF-β, transforming growth factor-β.

Assessment of the global variation using the PCA, a technique that emphasises variation and brings out strong patterns in a large dataset, indicated a molecular distinction between the primary BCs (red dots) that metastasise to the brain (BC_R) and the BCBMs (grey dots) (Figure 2A). A total of 272 genes were differentially expressed (DE) in primary BCs versus BCBM with Benjamin–Hochberg false discovery rate (FDR) <0.05 (Supplementary Table S1, available at https://doi.org/10.1016/j.esmoop.2022.100636). Of these, 112 genes were down-regulated, and 4 genes were up-regulated in BCBM with FDR <0.01 and log2 fold-change (log2 FC) >1.5 (Figure 2B, Supplementary Table S1, available at https://doi.org/10.1016/j.esmoop.2022.100636). Enrichment gene-set analysis (GSA), based on the DE data, grouped the down-regulated genes by biological functions and identified several dysregulated pathways, the top six of which are the matrix remodelling and metastasis, cytokine and chemokine signalling, lymphoid compartment, antigen presentation, immune cell adhesion and migration and co-stimulatory signalling. (Figure 2C). Genes enriched for metabolic stress, autophagy and epigenetic regulations were preserved between primary BC and BCBM (Figure 2C). The four genes up-regulated in BCBM in comparison to the primary BC were ARG2, SOX2, EGF and NCAM1 (FDR < 0.01 and log2 FC > 1.5, Supplementary Table S1, available at https://doi.org/10.1016/j.esmoop.2022.100636).

Several immuno-modulators and potential therapeutic targets such as PD-L1, CTLA4, TIGIT, CD27, CD276 (B7H3), CXCR4/CXCL12, CD73 (NT5E), CSF1/CSF1R and IDO1 were decreased in the BCBMs whereas the macrophage marker CD163, the microglia marker CX3CL1/CX3CR1, the pro-tumorigenic toll-like receptors (TLR1,2,5,7,8,9), the immune checkpoints programmed cell death protein 1 (PD-1) (PDCD1), CD47, STAT3 and the oncogenes MET, BIRC5 and LIF were not altered. The complete list of genes is presented in Supplementary Table S1, available at https://doi.org/10.1016/j.esmoop.2022.100636, and a selection of immune-oncology targets as identified in the Cancer Research Institute (CRI) iAtlas (https://www.cri-iatlas.org/) and Szekeley et al.^12^ is presented in Table 1.

**Table 1:** mRNA expression of immune-oncology targets in breast cancer brain metastasis

Gene	Gene family	Category	Function	Log2 FC	*P* value	BH *P* value
**IO targets decreased in breast cancer brain metastasis**						
CXCL12 (SDF1)	CXC chemokine	Ligand	Stimulatory	−2.38	2.01E−09	1.03E−07
CD27 (TNFRSF7)	TNFR	Receptor	Stimulatory	−2.56	2.03E−07	3.22E−06
CCL5 (RANTES)	Chemokine	Ligand	Stimulatory	−2.16	3.00E−07	4.40E−06
TGFB1	Cytokine	Ligand	Inhibitory	−1.18	5.04E−07	6.46E−06
SLAMF7	SLAM	Co-inhibitor	Inhibitory	−2.25	8.13E−07	9.50E−06
HLA-DRA	MHC class II	Antigen presentation		−1.58	1.16E−06	1.27E−05
CXCL9	CXC chemokine	Ligand	Stimulatory	−2.76	1.60E−06	1.59E−05
HLA-DPB1	MHC class II	Antigen presentation		−1.46	3.92E−06	3.58E−05
CD28	B7/CD28	Co-stimulator	Stimulatory	−1.89	4.72E−06	4.19E−05
HLA-DRB1	MHC class II	Antigen presentation		−1.69	7.94E−06	6.62E−05
ITGB2 (LFA1)	Integrin	Cell adhesion	Stimulatory	−1.23	1.10E−05	8.69E−05
CSF1	Cytokine	Other	Stimulatory	−1.6	1.50E−05	0.000114
PDCD1LG2 (PD-L2)	B7/CD28	Co-inhibitor	Inhibitory	−1.72	3.83E−05	0.000253
CD40	TNFR	Receptor	Stimulatory	−1.37	4.51E−05	0.000281
CCL4	Chemokine	Ligand	Stimulatory	−1.35	5.56E−05	0.000343
NT5E (CD73)	Enzyme	Other	Stimulatory	−1.47	8.46E−05	0.000481
HLA-DPA1	MHC class II	Antigen presentation		−1.3	0.000144	0.000765
GZMA	Granzyme	Other	Stimulatory	−1.8	0.000232	0.00114
JAK1	Enzyme	Kinase	Signalling	−0.508	0.000286	0.00138
TIGIT	PVR	Receptor	Inhibitory	−1.9	0.000289	0.00139
VISTA (B7-H5)	Immunoglobulin	Co-inhibitor	Inhibitory	−1.15	0.000306	0.00145
TLR4	Receptor	Receptor	Stimulatory	−0.86	0.000332	0.00156
CTLA4	Receptor	Receptor	Inhibitory	−1.46	0.000578	0.00247
IDO1	Enzyme	Other	Inhibitory	−1.97	0.0012	0.00474
CCR4	Chemokine	Receptor	Stimulatory	−1.47	0.00169	0.00627
CXCL10 (IP-10)	CXC chemokine	Ligand	Stimulatory	−1.6	0.00249	0.00896
CD276 (B7-H3)	B7/CD28	Co-inhibitor	Inhibitory	−0.543	0.00253	0.00906
LAG3	Immunoglobulin	Receptor	Inhibitory	−1.06	0.00467	0.0152
CXCR4	CXC chemokine	Receptor	Stimulatory	−0.684	0.00493	0.016
CSF1R	Immunoglobulin	Receptor	Stimulatory	−0.834	0.00526	0.0169
TNFRSF18 ( GITR)	TNFR	Receptor	Stimulatory	−1.1	0.0054	0.0171
HLA-DQA2	MHC class II	Antigen presentation		−1.31	0.00701	0.0212
IL2RA (CD122)	Cytokine receptor	Receptor	Stimulatory	−1.12	0.00818	0.0238
CCR5	Chemokine	Receptor	Stimulatory	−1.18	0.0114	0.0316
CD40LG	TNF	Ligand	Stimulatory	−1.15	0.0119	0.0328
CD274 (PD-L1)	B7/CD28	Co-inhibitor	Inhibitory	−1.09	0.0124	0.0336
KDR (VEGFR2)	Growth factor	Receptor	Inhibitory	−0.649	0.0131	0.0351
CCR2	Chemokine	Receptor	Stimulatory	−1.22	0.0141	0.037
CD70	TNF	Ligand	Stimulatory	−1.06	0.0196	0.0488
**IO targets increased in breast cancer brain metastasis**						
ARG2	Enzyme	Other	Inhibitory	1.99	3.41E−05	2.34E−04
NCAM1	Immunoglobulin	Cell adhesion	Stimulatory	2.1	0.00035	0.00162
SOX2	Transcription factor	Stemness	Inhibitory	3.76	6.71E−06	5.80E−05
**IO targets preserved in breast cancer brain metastasis**						
TNFRSF14 (HVEM)	TNFR	Receptor	Stimulatory	−0.472	0.0387	0.0839
CD80 (B7-1)	B7/CD28	Co-stimulator	Stimulatory	−0.786	0.0413	0.0882
LIF	Cytokine	Other	Inhibitory	0.781	0.062	0.123
ICAM1	Glycoprotein	Cell adhesion	Stimulatory	−0.71	0.0637	0.125
IL1B	Cytokine	Ligand	Stimulatory	−0.815	0.0837	0.161
PRF1	Pore	Other	Stimulatory	−0.932	0.119	0.209
HAVCR2 (TIM-3)	Immunoglobulin	Receptor	Inhibitory	−0.488	0.138	0.239
HLA-DQA1	MHC class II	Antigen presentation		−1.95	0.153	0.257
HLA-DQB1	MHC class II	Antigen presentation		−1.66	0.159	0.263
TLR8	Toll-like receptor	Receptor	Stimulatory	−0.522	0.186	0.298
VEGFA	Growth factor	Ligand	Inhibitory	0.361	0.211	0.331
PDCD1 (PD-1)	B7/CD28	Receptor	Inhibitory	−0.619	0.214	0.334
JAK2	Enzyme	Kinase	Signalling	−0.247	0.253	0.383
SIRPA	Immunoglobulin	Receptor	Inhibitory	−0.391	0.264	0.397
TNF	Cytokine	Ligand	Stimulatory	−0.514	0.291	0.422
TNFSF4 (OX40L)	TNF	Ligand	Stimulatory	−0.493	0.297	0.428
HMGB1	HMG-box	Other	Stimulatory	0.278	0.315	0.448
MET	Kinase	Receptor	Inhibitory	−0.451	0.335	0.465
TLR7	Toll-like receptor	Receptor	Stimulatory	−0.576	0.341	0.47
CX3CL1	CXC chemokine	Ligand	Stimulatory	−0.314	0.4	0.53
TLR2	Toll-like receptor	Receptor	Stimulatory	−0.201	0.425	0.549
TLR1	Toll-like receptor	Receptor	Stimulatory	−0.27	0.444	0.568
ADORA2A	Receptor	Receptor	Inhibitory	−0.261	0.483	0.603
VTCN1 (B7-H4)	B7/CD28	Co-inhibitor	Inhibitory	0.27	0.606	0.715
STAT3	Transcription factor	Other	Signalling	−0.0888	0.681	0.773
BIRC5	Antigen	Other	Inhibitory	0.112	0.718	0.801
CD47	Immunoglobulin	Ligand	Inhibitory	−0.102	0.72	0.802
ICOSLG (B7-H2)	Ligand	Co-stimulator	Stimulatory	−0.0582	0.755	0.83

Gene family, category and function of several immune-oncology (IO) targets (adopted from https://www.cri-iatlas.org/ and Szekeley et al.^12^) are presented along with the log2 fold-change (FC) in breast cancer brain metastasis relative to primary tumour, *P* values and Benjamin–Hochberg (BH) FDR-adjusted *P* values.

FDR, false discovery rate; MHC, major histocompatibility complex; SLAM, signalling lymphocytic activation molecule; TNF, tumour necrosis factor; TNFR, tumour necrosis factor receptor.

### Immune-related gene expression

Analysis of the prevalence of the different immune cell types in primary BCs that metastasised to the brain and their paired BCBMs showed marked differences in their immune profiles (Figure 2D). BCBMs lack natural killer cells (NK and NK56dim:effector) and have reduced dendritic cells (DCs) and mast cells in comparison to the primary BCs, whereas they have increased number of neutrophils, B cells and CD45 cells (Figure 2D). Tregs were low in both primary BCs and BCBMs. There was also an immune-related distinction of the primary BCs and BCBMs by immune cell type scoring as indicated in the dendrogram (Supplementary Figure S1, available at https://doi.org/10.1016/j.esmoop.2022.100636).

**Figure 3:**
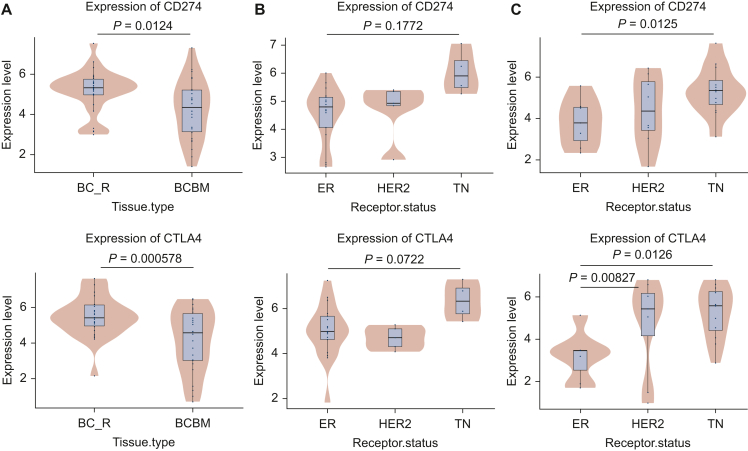
**PD-L1and CTLA4 transcript expression in BCBM.** (A) PD-L1 (CD274) and CTLA4 transcript expression (−log10 values) were reduced in BCBMs in comparison to the paired primary BCs that relapsed to the brain (BC_R). (B) In primary BCs, the TN cases showed higher PD-L1 and CTLA4 transcript levels compared to ER-positive and HER2-positive cases without reaching significance. (C) In BCBMs, PD-L1 (CD274) transcript expression was higher in the TN subtype whereas no difference was observed between ER-positive and HER2-positive subtypes. CTLA4 transcript expression was higher in both the HER2-positive and TN subtypes in comparison to the ER-positive subtype. BC, breast cancer; BCBM, breast cancer brain metastasis; ER, estrogen receptor; HER2, human epidermal growth factor receptor 2; PD-L1, programmed death-ligand 1; TN, triple negative.

Differences in PD-L1 (CD274) and CTLA4 transcript expression (−log10 values) were also observed between BCs that relapsed to the brain (BC_R) and their paired BCBMs (Figure 3A). Expression of both PD-L1 (CD274) and CTLA4 was higher in the primary BCs than in the BCBM (*P* = 0.0124 and *P* < 0.001, respectively). Analysis of PD-L1 (CD274) and CTLA4 transcript expression based on receptor subtype in primary BC demonstrated an increased expression in the TNBC cases but did not reach statistical significance (*P* = 0.1772 and *P* = 0.0722, respectively, Figure 3B) due to the low number of TNBC cases. When comparing BCBM based on these subtypes, the expression of PD-L1 (CD274) and CTLA4 was significantly higher in TN cases as compared to ER-positive cases (*P* = 0.0125 and *P* = 0.0126, respectively). There was also a significantly higher CTLA4 expression in HER2-positive BCBM compared to those that were ER positive (*P* < 0.01) (Figure 3C).

### ARG2 expression

**Figure 4:**
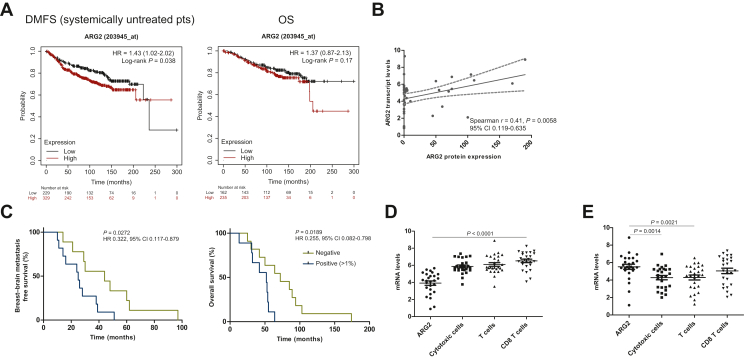
**ARG2 expression and its clinical association.** (A) KM plotter analysis showed that high mRNA expression of ARG2 correlated with worse distant metastasis-free survival (DMFS) but not overall survival (OS). (B) Spearman’s correlation *r* showed a significant positive correlation between ARG2 mRNA and protein expression. (C) ARG2 protein-positive expression (>1% ARG2 staining of tumour cells) in primary BCs correlated with worse breast–brain metastasis-free survival (BMFS) (time between breast–brain diagnosis/surgery) and OS. (D, E) Wilcoxon signed rank *t*-test (Gaussian approximation) showed a significant difference between the transcript levels of ARG2 and cytotoxic cells, T cells and CD8 cells in (D) primary BCs (*P* < 0.0001 for all comparisons) and (E) between the transcript levels of ARG2 and cytotoxic cells and T cells but not CD8 cells in BM (*P* = 0.0014, *P* = 0.021, *P* = 0.3219, respectively). BC, breast cancer; BM, brain metastasis; CI, confidence interval; HR, hazard ratio; KM, Kaplan–Meier; mRNA, messenger RNA.

ARG2 transcript profiling demonstrated higher expression in BCBMs than in primary BCs (FDR < 0.01 and log2 FC > 1.5). Data from KM plotter analysis software (www.kmplot.com)^23^ demonstrated that high mRNA expression of ARG2 correlated with worse DMFS [*P* = 0.038, hazard ratio (HR) 1.43, 95% confidence interval (CI) 1.02-2.02] but not OS (*P* = 0.17, HR 1.73, 95% CI 0.87-2.13) in patients not treated with systemic therapy (Figure 4A). ARG2 expression by IHC was cytoplasmic, dot-like or coarsely granular in nature (Supplementary Figure S2, available at https://doi.org/10.1016/j.esmoop.2022.100636), with 50% (11 of 22) of BCs and 63.6% (14 of 22) of BCBMs being defined as ARG2 positive (>1% ARG2 staining of tumour cells).^22^ No ARG2 expression was observed in the primary BCs (*n* = 9) that did not develop BMs (median follow-up 15.6 years). A positive correlation between protein and mRNA transcript levels of ARG2 as identified by the nCounter™ IO360 expression assay was observed (Spearman’s *r* = 0.41, *P* = 0.0058, 95% CI 0.119-0.635, Figure 4B). The expression of ARG2 protein in primary BCs that metastasised to the brain was associated with a significantly worse BMFS (*P* = 0.027, HR 0.332, 95% CI 0.117-0.879, Figure 4C) and OS (*P* = 0.019, HR 0.255, 95% CI 0.082-0.798, Figure 4C). There was no association of ARG2 protein expression between time of neurosurgery and time to death (*P* = 0.643, HR 0.793, 95% CI 0.294-2.128).

Wilcoxon signed rank *t*-test (Gaussian approximation) demonstrated a significant difference between the transcript levels of ARG2 and cytotoxic cells, T cells and CD8 T cells in primary BCs (*P* < 0.0001 for all comparisons, Figure 4D), with higher transcript levels of T cells being associated with lower ARG2. Similarly, there was a significant difference between the transcript levels of ARG2 and cytotoxic cells and T cells (*P* = 0.0014, *P* = 0.021, Figure 4E) but not CD8 T cells in BCBM (*P* = 0.3219, Figure 4E) with higher ARG2 and lower T-cell transcript levels.

## Discussion

With the development of immunotherapy for BC, several studies have explored differences in the tumour-immune microenvironment between the primary and metastatic site.^12^, ^13^, ^14^, ^15^, ^16^, ^17^ Differences within the tumour-immune microenvironment have been identified during the progression from *in situ* to invasive disease, between the BC subtypes and have been linked to prognosis and clinical response.^24^, ^25^, ^26^, ^27^ MBC has been shown to have a different immune tumour microenvironment from the primary BCs, including a lower TIL content and PD-L1 positivity compared to primary BCs as well as being more immunologically inert.^12^, ^13^, ^14^, ^15^, ^16^^,^^28^

In this study, assessment of the global variation using PCA indicated a molecular distinction between the primary BCs that metastasise to the brain (BC_R) and the BCBMs highlighting differences in their tumour-immune microenvironments and that DE is associated with cancer tissue type. A significant reduction of TILs in BCBMs in comparison to the primary BCs was observed by assessing the H&E sections and by the NanoString™ TIL immunoscore. This observation agrees with a previously reported study assessing TILs in 46 cases of BCBMs^17^ and several studies assessing TILs in other metastatic sites including a limited number of BCBMs.^12^, ^13^, ^14^, ^15^, ^16^ Despite this, 3.8% of BCBMs were identified as hot (H&E assessment). Similarly, transcript expression of both PD-L1 (CD274) and CTLA4 was higher in the primary BCs than in the BCBM (*P* = 0.0124 and *P* < 0.001, respectively) consistent with the observed TIL data. However, difference by subtypes was observed in the primary BC with both PD-L1 (CD274) and CTLA4 demonstrating increased expression in the TNBC cases which did not reach significance likely due to the small number of cases (*n* = 4). Within the BCBM, PD-L1 transcripts were significantly higher in the TN subtype with CTLA4 transcript expression significantly higher in both TN and HER2-positive subtypes. We have previously demonstrated a significant increase in PD-L1 amplification and protein expression in TN BCBM.^29^^,^^30^ The clinical benefit of immunotherapy for CNS disease has been demonstrated for more immunogenic cancers such as melanoma and lung cancer.^31^^,^^32^ Taken together, this clinical data, alongside our previous demonstrating an increase in PD-L1 amplification and protein expression in TN BCBM,^29^^,^^30^ and the current data support exploring the CNS activity of immunotherapy in TN BCBM enriching for those cases which express PD-L1 and/or CTLA4.^33^^,^^34^ However, keeping in mind the complexity of tumour-immune microenvironment of BMs, additional gene targets and immune checkpoint regulators/inhibitors should be explored.^15^, ^16^, ^17^, ^18^, ^19^^,^^29^^,^^30^

Gene expression profiling has demonstrated the immune depleted environment of BCBM (Supplementary Table S1, available at https://doi.org/10.1016/j.esmoop.2022.100636, Table 1) with genes involved in matrix remodelling and metastasis, cytokine–chemokine signalling, lymphoid compartment, antigen presentation and immune cell adhesion and migration decreased in BCBM compared to primary BCs. The data highlight the importance of developing therapeutic strategies that block this immune suppression in order to potentially sensitise BCBMs to immunotherapy. However, the expression of immune-oncology therapeutic targets such as PD-1 (PDCD1), STAT3 and CD47 and oncogenes such as BIRC5 and MET were preserved in BCBMs and we previously demonstrated that cMET protein is highly expressed in primary BCs relapsing to the brain and their paired BMs.^19^ Given this, combining inhibitors of BIRC5 or MET with currently available immunotherapy agents represents a rationale combination strategy to explore in the treatment of BCBM.

Only four genes, ARG2, SOX2, EGF and NCAM1, were significantly up-regulated in BCBM compared to the primary BCs (Supplementary Table S1, available at https://doi.org/10.1016/j.esmoop.2022.100636, Table 1). We further investigated ARG2 (mitochondrial arginase 2) as it is the main arginase isoform in BC^35^^,^^36^ and is overexpressed in glioblastoma,^37^^,^^38^ the most common and aggressive primary brain tumour in adults. ARG2 has an immunosuppressive role given that it induces extracellular arginine depletion which can modulate T-cell function.^39^^,^^40^ In addition, a study showed that microRNA-155 (miR155)-deficient mice DCs had elevated levels of Arg2 impairing T-cell proliferation whereas overexpression of miR155 inhibited Arg2 expression establishing an arginine-rich microenvironment, permissive for T-cell proliferation.^41^ Consistent with these data, we have previously identified reduced expression of miR155 in BCBMs in comparison to their paired primary BCs^19^ and in this study we observed up-regulated expression of ARG2 in BCBMs. In keeping with the potential importance of T cells in the context of BM, the survival of patients with BM has been associated with T-cell densities, with higher density associated with a better outcome.^42^

Utilising data from the KM plotter analysis software (www.kmplot.com),^23^ we demonstrated that high mRNA expression of ARG2 correlated with worst DMFS but not OS and showed a positive correlation between mRNA and protein expression. ARG2 protein expression was identified in 50% of BCs and 63.6% of BCBMs whereas there was no detectable ARG2 expression in a small cohort of primary BCs that did not develop metastatic disease. We also demonstrate that BC patients positive for ARG2 had worse BMFS and OS, highlighting the clinical importance of ARG2 in aggressive BC and potentially in the development of BCBM. Our data agree with other studies showing the prognostic potential of ARG2,^22^^,^^43^, ^44^, ^45^ while a recent study showed that ARG2 promotes melanoma progression and metastasis through STAT3 signalling, also involved in BM.^46^^,^^47^ Since arginase has been identified as a potential biomarker of disease progression, investigating the therapeutic efficacy of arginase inhibitors as monotherapy or in combination with PD-1/PD-L1 inhibitors has been of great research interest^38^^,^^40^^,^^48^, ^49^, ^50^, ^51^, ^52^ and a number of clinical trials are ongoing (Supplementary Table S2, available at https://doi.org/10.1016/j.esmoop.2022.100636) in advanced solid tumours and glioblastoma. Finally, we observed that there was a significant difference between the transcript levels of ARG2 and cytotoxic cells and T cells in both BC and BCBM samples (Figure 4) indicative of a depleted T-cell response.

This study is not without limitations. These include the archival nature of the samples with the limited clinico-pathological information, the small number of TN cases within the primary BCs and the current lack of confirmation of findings in an independent study. However, a prospective collection of BCBM in the UK is currently underway (CNS PRIMROSE; ISRCTN18204314) which will provide access to a large number of prospectively collected annotated pairs of primary BCs and BCBMs enabling a better characterisation of the tumour and immune microenvironment. In addition, the importance of ARG2 is based on transcript and protein expression in clinical material, whereas functional studies will allow us to elucidate further its role in BCBM and to test new immunotherapy approaches.

In summary, we confirmed that BCBMs are immunologically more inactive than primary BCs as demonstrated by decreased TIL content and down-regulation of major immune-related pathways. However, PD-L1 and CTLA4 transcripts were high in TN subtypes. We also showed preservation or increases in a subset of immuno-oncology-related genes. Finally, we showed that ARG2 can influence the immune microenvironment and is associated with poor clinical outcomes. Given these data, studies investigating novel immune checkpoint inhibitors and/or immunotherapy approaches for BCBM are warranted.

## Supplementary data

Supplementary Figure 1. Immune cell type score. Immune cell type score by nCounter advanced analysis showed a separation of primary BCs and BCBMs highlighting the differences in their immune environment. **Supplementary Figure 2.** ARG2 staining by immunohistochemistry. A. Representative examples stained for ARG2 of a primary non-metastatic breast cancer, a breast cancer case that developed brain metastasis and its matched brain metastasis sample. The images are presented at x10 resolution to show the extent of cytoplasmic staining and x40 to visualise better the dot-like/coarsely granular staining. A prostate sample used as a positive control is also presented showing strong ARG2 staining.
