# Supplementary material for: Characterisation of the immune microenvironment of primary breast cancer and brain metastasis reveals depleted T-cell response associated to ARG2 expression
Source: ESMO Open. 2022 Nov 21;7(6):100636. doi: 10.1016/j.esmoop.2022.100636 (PMC9808462; doi:10.1016/j.esmoop.2022.100636)
Supplement: Supplementary Figure 1. Immune cell type score. — Immune cell type score by nCounter advanced analysis showed a separation of primary BCs and BCBMs highlighting the differences in their immune environment. Supplementary Figure 2. ARG2 staining by immunohistochemistry. A. Representative examples stained for ARG2 of a primary non-metastatic breast cancer, a breast cancer case that developed brain metastasis and its matched brain metastasis sample. The images are presented at x10 resolution to show the extent of cytoplasmic staining and x40 to visualise better the dot-like/coarsely granular staining. A prostate sample used as a positive control is also presented showing strong ARG2 staining. [file mmc1.pptx]

## Slide 1
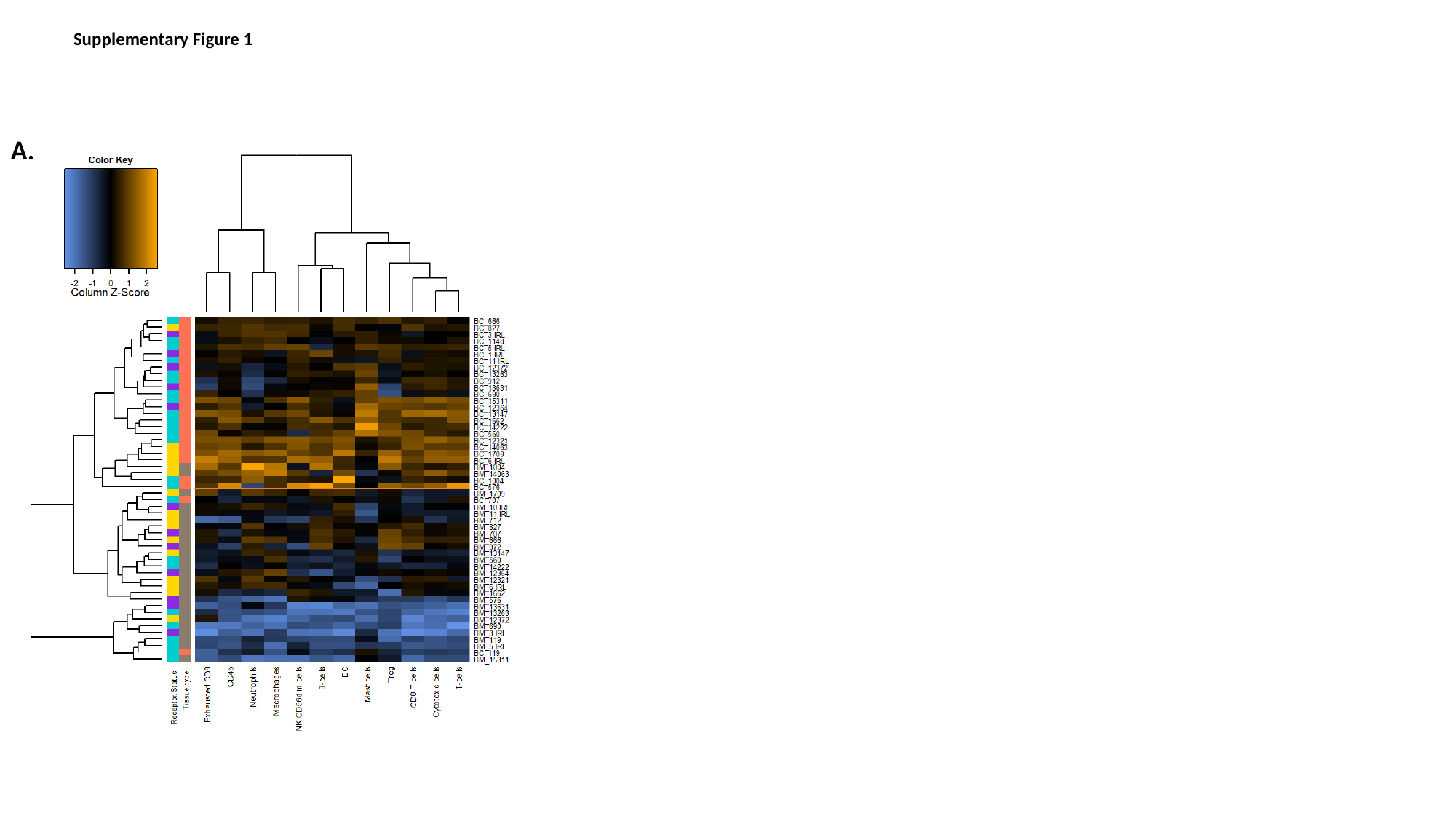

Supplementary Figure 1
A.

## Slide 2
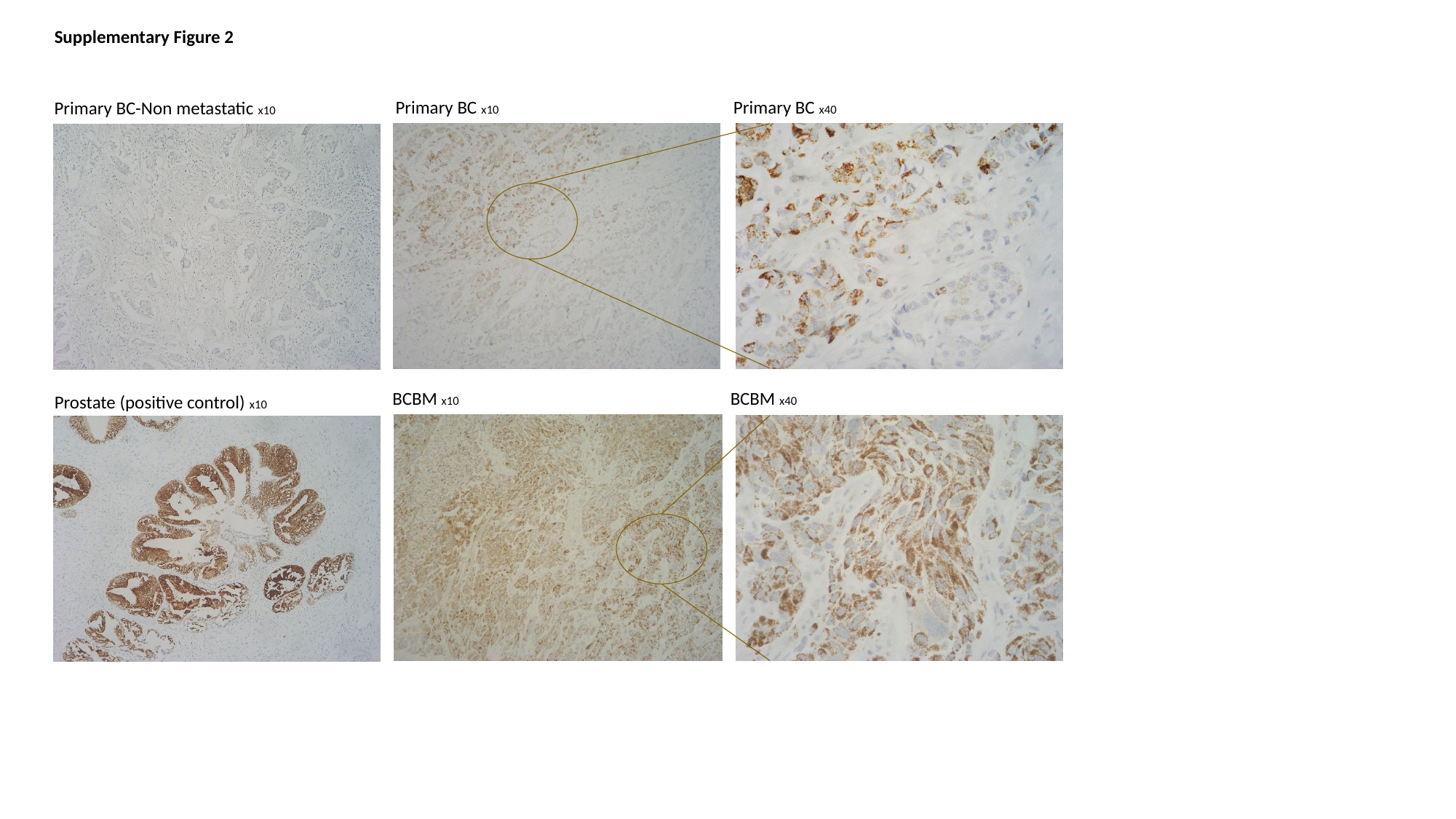

Supplementary Figure 2
Primary BC x10
Primary BC x40
Primary BC-Non metastatic x10
BCBM x10
BCBM x40
Prostate (positive control) x10
