# Supplementary material for: Characterisation of the immune microenvironment of primary breast cancer and brain metastasis reveals depleted T-cell response associated to ARG2 expression
Source: ESMO Open. 2022 Nov 21;7(6):100636. doi: 10.1016/j.esmoop.2022.100636 (PMC9808462; doi:10.1016/j.esmoop.2022.100636)
Supplement: Supplementary Tables S1 and S2 [file mmc2.docx]

**Supplementary Table 1: Patient characteristics and inclusion into the study.** The table summarises the receptor status of the patients in the primary BC and in BM, TILs assessment by H&E and inclusion into the nCounter IO360 assay and analysis.

| **Patient N^o^** | **Status** | **ER status of the 1^o^BC** | **ER status of**  **BM** | **PgR status of the 1^o^BC** | **PgR status of**  **BM** | **HER2 status of the 1^o^BC** | **HER2 status of**  **BM** | **Time (months) between breast-brain surgery /recurrence** | **Time (months) between breast surgery and death** | **Time (months) between brain surgery and death** | **% TILs (H&E)**  **and immunoscore in the 1^o^BC** | | **% TILs (H&E)**  **and immunoscore in the BM** | | **nCounter IO360 assay**  **1^o^BC BM** | |
| --- | --- | --- | --- | --- | --- | --- | --- | --- | --- | --- | --- | --- | --- | --- | --- | --- |
| 119 | ER+/HER2- | + | + | - | + | - | - | 62 | 85 | 23 | 0% | Cold | 0% | Cold | √ | √ |
| 560 | ER+/HER2- | + | + | + | + | - | - | 97 | 103 | 6 | 15% | Altered: IS | 5% | Cold | √ | √ |
| 666 | ER+/HER2- | + | - | + | - | - | - | 38 | 55 | 17 | 5% | Cold | 8% | Cold | √ | √ |
| 690 | ER+/HER2- | + | + | + | + | - | - | 44 | 72 | 28 | 3% | Cold | 1% | Cold | √ | √ |
| 707 | ER+/HER2+ | + | - | - | - | + | + | 48 | 89 | 41 | 12% | Altered: IS | 3% | Cold | √ | √ |
| 712 | ER+/HER2- | + | - | - | - | - | - | 28 | 32 | 4 | 3% | Cold | 8% | Cold | √* | √ |
| 756 | ER+/HER2+ | + | - | - | - | + | + | 25 | 53 | 30 | 0% | Cold | 1% | Cold | √ | √ |
| 827 | TN | - | - | - | + | - | - | 16 | NA | NA | 15% | Altered: E | 3% | Cold | √ | √ |
| 912 | ER+/HER2- | + | + | + | + | - | - | 60 | 94 | 34 | 25% | Altered: IS | 2% | Cold | √ | √ |
| 972 | ER+/HER2+ | + | - | + | + | + | + | 27 | 29 | 2 | - | - | NA | | - | √* |
| 1004 | ER+/HER2- | + | - | - | + | - | - | 10 | NA | NA | 45% | Hot | 15% | Altered: IS | √ | √ |
| 1148 | ER+/HER2- | + | + | + | + | - | - | 51 | 52 | 1 | 12% | Altered: E | 45% | Hot | √ | √* |
| 1662 | ER+/HER2- | + | - | - | + | - | - | 39 | 54 | 15 | 12% | Altered: E | 15% | Altered: IS | √ | √ |
| 1709 | TN | - | - | - | + | - | - | 11 | 13 | 2 | 20% | Altered: IS | 3% | Cold | √ | √ |
| 12321 | ER+/HER2- | + | - | - | - | - | - | 14 | 64 | 50 | 25% | Altered: IS | 5% | Cold | √ | √ |
| 12372 | ER-/HER2+ | - | NA | - | NA | + | + | 14 | 25 | 11 | 5% | Cold | 2% | Cold | √ | √ |
| 12364 | ER-/HER2+ | - | - | - | - | + | + | 29 | 64 | 35 | 20% | Altered: IS | 12% | Altered: IS | √ | √ |
| 13147 | ER+/HER2- | + | - | + | - | - | - | 24 | 42 | 18 | 20% | Altered: IS | 8% | Cold | √ | √ |
| 13263 | ER+/HER2- | + | + | - | - | - | - | 30 | 42 | 12 | 5% | Cold | 3% | Cold | √ | √ |
| 13631 | ER-/HER2+ | - | - | - | NA | + | + | 21 | 30 | 9 | 5% | Cold | 3% | Cold | √ | √ |
| 14063 | TN | - | - | - | NA | - | - | 26 | 31 | 5 | NA | | 35% | Altered: IS | √ | √ |
| 14222 | ER+/HER2- | + | + | + | + | - | - | Synchronous | 50 | 50 | 8% | Cold | 15% | Altered: IS | √ | √ |
| 15311 | ER+/HER2- | + | + | + | + | - | - | NA | 174 | NA | 45% | Hot | 5% | Cold | √ | √ |
| RCSI_1 | ER-/HER2+ | - | - | - | - | + | + | 20 | 31 | 11 | 8% | Cold | - | - | √ | - |
| RCSI_3 | ER-/HER2+ | - | - | - | - | + | + | 67 | 115 | 48 | 15% | Altered: IS | 3% | Cold | √ | √ |
| RCSI_5 | ER+/HER2- | + | + | - | - | - | + | 53 | 74 | 21 | NA | | 5% | Cold | √ | √ |
| RCSI_6 | TN | - | - | - | - | - | - | 23 | 40 | 17 | 20% | Altered: IS | 5% | Cold | √ | √ |
| RCSI_10 | ER-/HER2+ | - | - | - | - | + | + | 70 | 112 | 42 | - | - | 5% | Cold | - | √ |
| RCSI_11 | ER+/HER2- | + | - | - | - | - | - | 8 | 17 | 9 | 45% | Hot | 35% | Altered: IS | √ | √ |
| NA: Not assessable, IS: Immunosuppressed, E: Excluded, (-) not available, (*) Cases that failed Quality control and were excluded from the nCounter 4.0 Advanced analysis. | | | | | | | | | | | | | | | | |

**Supplementary Table 2. Clinical Trials using Arginase inhibitors.** Current clinical trials using Arginase inhibitors with/without immune checkpoint inhibitors or chemotherapy as identified in ClinicalTrials.gov in advanced solid tumours and in glioblastoma.

| **Clinical Trial Id.** | **Study** | **Drugs** |
| --- | --- | --- |
| NCT03455140 | A Study Evaluating the Safety and Activity of Pegylated Recombinant Human Arginase (BCT-100) (PARC) | PEG-BCT-100 |
| NCT02903914 | Arginase Inhibitor INCB001158 as a Single Agent and in Combination With Immune Checkpoint Therapy in Patients With Advanced/Metastatic Solid Tumors | INCB001158, Pembrolizumab |
| NCT03910530 | A Study of INCMGA00012, INCB001158, and the Combination in Japanese Participants With Advanced Solid Tumors | INCB001158, Retifanlimab |
| NCT03314935 | A Phase 1/2 Study of INCB001158 in Combination With Chemotherapy in Subjects With Solid Tumors | INCB001158, Oxaliplatin, Leucovorin, Cisplatin, Paclitaxel, 5-Fluorouracil, Gemcitabine, |
| NCT02017249 | Efficacy Study of Oral Arginine to Improve Immune Function in Glioblastoma Multiforme (ArginineGBM) | Oral Arginine powder |
| NCT04587830 | Phase 1B Trial of ADI-PEG 20 Plus Radiotherapy and Temozolomide in Subjects With Newly Diagnosed Glioblastoma Multiforme | ADI-PEG20, Temozolomide |
